# Interferon-Gamma Improves Macrophages Function against* M. tuberculosis* in Multidrug-Resistant Tuberculosis Patients

**DOI:** 10.1155/2016/7295390

**Published:** 2016-07-12

**Authors:** Taj Ali Khan, Humaira Mazhar, Shamim Saleha, Hamid Nawaz Tipu, Niaz Muhammad, Muhammad Nasser Abbas

**Affiliations:** ^1^Department of Microbiology, Kohat University of Science and Technology, KPK, Kohat 26000, Pakistan; ^2^Department of Biotechnology, Kohat University of Science and Technology, KPK, Kohat 26000, Pakistan; ^3^Department of Immunology, Armed Forces Institute of Pathology, Rawalpindi 46000, Pakistan

## Abstract

*Background*.* Mycobacterium tuberculosis* (*M. tuberculosis*) that causes tuberculosis (TB) kills millions of infected people annually especially multidrug-resistant tuberculosis (MDR-TB). On infection, macrophages recognize the mycobacteria by toll-like receptor (TLR) followed by phagocytosis and control of mycobacteria. In addition, macrophages also secrete IL-12 to induce IFN-*γ* production by T, which, in turn, increases the phagocytosis and oxidative burst. Individuals with defects in innate or adaptive immunity exhibit increased susceptibility to* M. tuberculosis*. Understanding these immunologic mechanisms will help in TB control. We aimed to investigate the immunopathologic mechanisms in MDR-TB and role of recombinant human interferon-gamma (rhIFN-*γ*).* Study Design and Methods*. Monocyte-derived macrophages (MDMs) were generated from peripheral blood mononuclear cells of MDR-TB patients and healthy subjects and were investigated for immunologic response by ELISA and flow cytometry.* Results*. Different functional and molecular anomalies were observed in macrophages. In addition, a defective immune response to* M. tuberculosis* from the patient's MDMs was characterized, which in turn improved by pretreatment with rhIFN-*γ*.* Conclusion*. This work highlights the fact that rhIFN-*γ* improves macrophages function against* M. tuberculosis* and treatment of patients with poor responsiveness to TB therapy may be needed in future to include IFN-*γ* as adjuvant therapy after the full characterization of pathological and molecular mechanisms in these and in other more multidrug-resistant TB patients.

## 1. Introduction

The genus* Mycobacterium* contains several species: many of which cause human diseases.* Mycobacterium tuberculosis* and three closely related* mycobacterium* species (*M. bovis*,* M. africanum*, and* M. microti*) cause tuberculosis disease, composing what is known as* Mycobacterium tuberculosis complex* [1]. One-third of the world's population is infected by* M. tuberculosis*, while only about 5% of infected individuals develop the disease within the first year of infection and another 5% develop the disease later in life, indicating remarkable individual differences which may be related to multifactors including immune system [2, 3]. Tuberculosis (TB) remains a scourge of humanity and a major global health problem and the global prevalence of* M. tuberculosis* infection is about 32% [4, 5]. Effective TB control will require a deeper understanding of the impact of drug resistance on the host-pathogen interaction and of the immune system underlying the relative success of drug-resistant strains [6].

The course of mycobacterium infections is dependent on the interactions of the* Mycobacterium* and the immune response of the host. Upon the first encounter with mycobacteria, the innate arm of the immune system is rapidly activated and the adaptive arm of the immune system synergistically cooperates to control the mycobacteria growth and spreading. Elimination of* M. tuberculosis* infection mainly depends on the success of the interaction between infected macrophages and T lymphocytes despite the involvement of other cells [7]. Macrophages are important effector cells in immunity against intracellular bacteria. On infection, macrophages (MO) recognize the mycobacteria by toll-like receptor (TLR) engagement (especially TLR1/2 and TLR2/6) followed by phagocytosis and control of mycobacteria growth. In addition, macrophages and dendritic cells also secrete cytokines such as IL-12 and IL23 to induce IFN-*γ* production by T and NK cells, which, in turn, increases the phagocytosis, phagolysosomal fusion, oxidative burst, and other not fully clear nonoxidative mechanisms [8]. For an efficient T helper 1 (Th1, IFN-*γ* producer cells) differentiation, costimulation (e.g., CD40L-CD40 and CD28-CD80/CD86 interactions) and NEMO/NF-*κ*B dependent signaling [9] are required. On the other hand, the negative regulation of IFN-*γ* production involves different mechanism, including production of Th2 cytokines (IL-4, IL-5, and IL-13) [10] and the participation of suppressor of cytokine signaling- (SOCS-) 1. In accordance, patients with severe pulmonary tuberculosis have been reported to have skewed Th1 to Th2 immune response [11] and raised SOCS-1 levels [12].

Individuals with defects in the innate or adaptive immunity affecting the above described mechanisms exhibit increased susceptibility to* M. tuberculosis* [13] and also need to investigate such immune response in MDR-TB patients which may be a key determinant. Here, we aimed to investigate the immunopathological mechanisms in MDR-TB patients and the effect of rhIFN-*γ* against the control of* M. tuberculosis* by macrophages.

## 2. Materials and Methods

### 2.1. Patients and Controls

Relevant clinical data of MDR-TB patients from consanguineous families confirmed by drug susceptibility were obtained from the medical records (Table 1). Signed consent forms were obtained from all patients or their parents, and blood samples were collected under institutional guidelines. The study was approved by the Departmental Ethics Committee at the Department of Microbiology, Kohat University of Science and Technology, Pakistan, according to the Helsinki Convention.

### 2.2. Generation of Monocyte-Derived Macrophages

Human MDMs were obtained as described by Esquivel-Solís et al. [14]. Briefly, PBMCs were isolated from heparinized blood after Ficoll-Hypaque sedimentation and adherent monocytes were cultured at 37°C in a humidified 5% CO_2_ atmosphere for 7 days in the presence of 5 ng/mL of macrophage colony stimulating factor (M-CSF, Peprotech).

### 2.3. Investigation of Cytokines Production

MDMs were analyzed for the production of IL-12, TNF-*α*, and IL-6 as previously described [15]. Briefly, cells were activated at 37°C in 5% CO_2_, in 96-well round-bottom plates (Greiner Bio-One, Frickenhausen, Germany) in a final volume of 200 *µ*L of RPMI 1640, supplemented with 10% FCS. The production of IL-12, TNF-*α*, and IL-6 was measured after 24 hours of incubation with IFN-*γ* (200 IU/mL, Imukin®, Boehringer Ingelheim) or/and live BCG (1 MDM/10 BCG), respectively. BCG was cultured as previously described [16]. The production of IL-12, TNF-*α*, and IL-6 in the supernatants was analyzed by ELISA according to the manufacturer's instructions (Becton Dickinson).

### 2.4. Dihydrorhodamine-123 (DHR) Assay

Oxidative burst in MDMs was assessed as previously described [17]. MDMS were incubated for 5 min at 37°C with dihydrorhodamine 123 (Sigma-Aldrich) after activation for 1 hour with phorbol myristate acetate (PMA, 300 ng/mL, Sigma Laboratories, St. Louis, MO, USA) and the data obtained were analyzed by using FlowJo software (Treestar, Inc., Ashland, Ore).

### 2.5. Flow Cytometric Analysis of Surface Expression of Receptors

We determined the expression of specific surface molecules functioning known as receptors (TLR2, TLR4, TLR10, and IFN-*γ*R1) on MDMs and (IL12R*β*1 and IL12R*β*2) on lymphocytes in relation to possible anomalies in these signaling pathways. For this, cells were incubated with specific monoclonal antibodies at 4°C for 30 min in the dark. After incubation, cells were washed twice with PBS and the cells were fixed in PBS plus 1% paraformaldehyde. The protein surface expression was analyzed by flow cytometry on a BD FACSCanto II Cytometer and the data obtained were analyzed by using FlowJo software (Treestar, Inc., Ashland, Ore).

### 2.6. Phagocytosis and Growth Control of* M. tuberculosis* by MDMs

The analysis of* M. tuberculosis* (H37Rv strain) phagocytosis and growth control by MDMs was carried out as previously described [18]. In brief, MDMs were challenged at ratio 1/1 (*M. tuberculosis*/MDMs) during 3 h (day 0) and washed to remove extracellular mycobacteria. On day 0 and after 6 days, the MDMs were lysed with 0.1% saponin treatment, and the homogenates were diluted and plated in Middlebrook 7H10 medium supplemented with 10% OADC (Difco; acid/albumin/dextrose/catalase). The resultant colonies were assessed after 21 days of incubation at 37°C.* M. tuberculosis* uptake (phagocytosis index) data were obtained from the CFU counts performed on day 0, and the* M. tuberculosis* growth index was determined based on the ratio of the CFU numbers on day 6 to the CFU number on day 0.

### 2.7. Statistical Analysis

Statistical significance was assessed by the nonparametric Mann-Whitney test. Data were expressed as median and 25th and 75th percentiles. The statistical analyses were performed using GraphPad PRISM 4.03 software (GraphPad Software, San Diego, CA, USA) and differences with *P* ≤ 0.05 were considered significant.

## 3. Results

### 3.1. Reduced Production of IL-12, TNF-*α*, and IL-6 by Patient's MDMs

**Figure 1:**
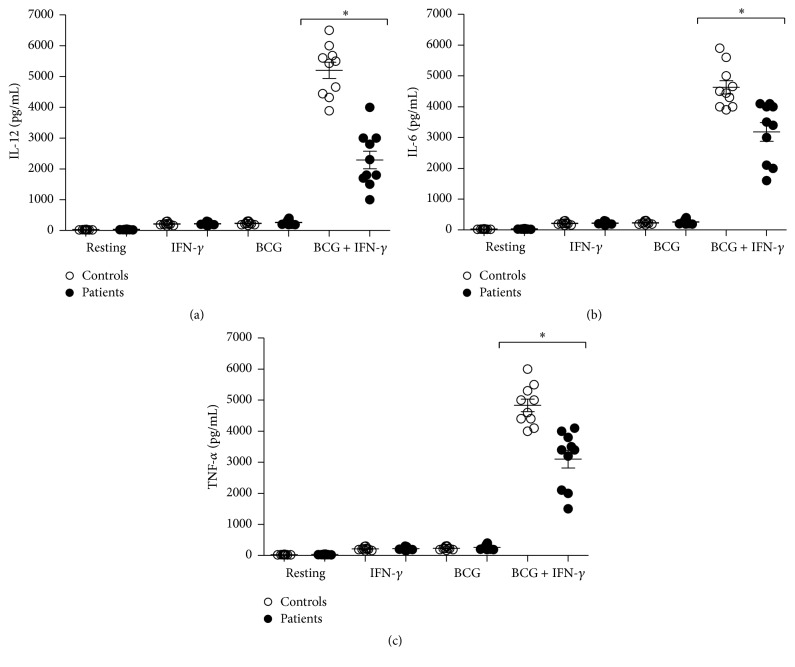
Investigation of cytokines production. For the cytokines release, MDMs were stimulated by IFN-*γ*, BCG, or BCG + IFN-*γ* and analyzed by ELISA (a) IL-12 (b), IL-6 (c), and TNF-*α*. Significant difference is denoted by asterisk (*P* ≤ 0.05; *n* = 10 patients and 10 controls; Mann-Whitney test).

IL-12, TNF-*α*, and IL-6 cytokines secreted by macrophages are important against the control of mycobacteria; therefore, we decided to evaluate IL-12, TNF-*α*, and IL-6. We compared the production of IL-12, TNF-*α*, and IL-6 after stimulation with BCG alone, BCG plus IFN-*γ*, and MDMs from the patients group produced reduced amounts of IL-12, TNF-*α*, and IL-6 with statistic difference in comparison to MDMs from healthy controls (Figures 1(a)–1(c)).

### 3.2. Impaired Production of Oxidative Burst by Patient's MDMs Improves with Exogenous IFN-*γ*

**Figure 2:**
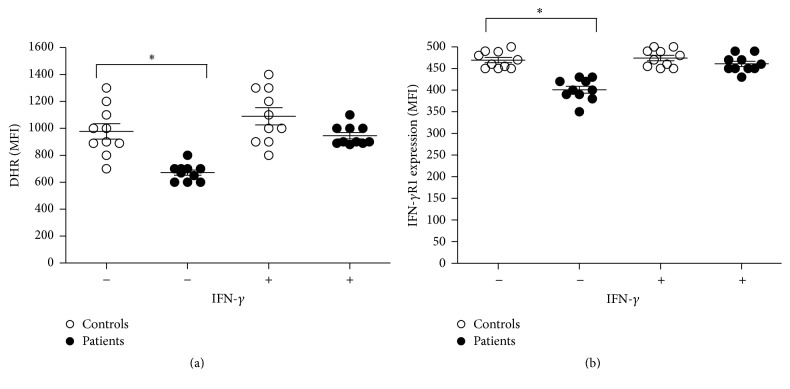
(a) MDMs remained untreated or were treated for 24 h with rhIFN-*γ* (100 U/mL) and the respiratory burst of MDMs was induced by PMA (90 mM) and analyzed by flow cytometry. (b) IFN-*γ*R1 surface expression on MDMs was performed by flow cytometry. Significant difference is denoted by asterisk (*P* ≤ 0.05; *n* = 10 patients and 10 controls; Mann-Whitney test).

Next, we investigated our cohort for oxidative burst by DHR assay. MDMs from patients showed impaired oxidative burst in response to PMA in comparison to healthy controls. On the other hand, IFN-*γ* pretreated MDMs from patients and healthy control subjects displayed similar oxidative burst response in comparison suggesting that IFN-*γ* improve the MDMs function (Figure 2(a)).

### 3.3. Defective Surface Expression of IFN-*γ*R1 on MDMs Revered with Exogenous IFN-*γ*

Because TLR2, TLR6, IFN-*γ*R, and IL12R signaling play important role in immune response against mycobacteria, we evaluated their expression on MDMs and T cells by flow cytometry using specific antibodies. MDMs from patients with MDR-TB showed reduced levels of IFN-*γ*R1 expression (Figure 2(b)), while normal surface expression of TLR2 and TLR6 (data not shown) compared with normal controls and similar T cells from patients showed similar IL12R*β*1 expression compared to control subjects. More importantly, addition of IFN-*γ* to the culture unregulated IFN-*γ*R1 molecules on MDMs from patients to normal levels.

### 3.4. Analysis of Phagocytosis and Mycobacterial Growth inside Monocytes-Derived Macrophages

**Figure 3:**
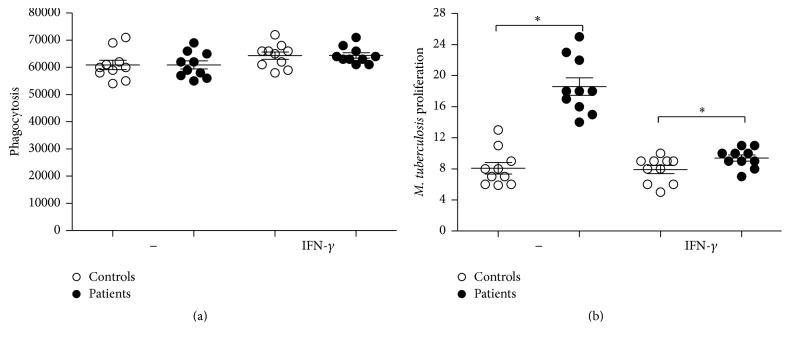
Prior to being assayed, MDMs remained without (−) or were treated for 24 h with (+) rhIFN-*γ* (100 U/mL). Data were obtained from the CFU counts performed on day 0, and the* M. tuberculosis* growth index was determined based on the ratio of day 3 to day 0. Significant difference is denoted by asterisk (*P* ≤ 0.05; *n* = 10 patients and 10 controls; Mann-Whitney test).

Considering the activating properties of rhIFN-*γ* on phagocytes and its beneficial therapeutic effect for patients with susceptibility to mycobacterial infections [19], we assessed the capacity of macrophages from our patient to phagocytose and control the proliferation of* M. tuberculosis* before and after rhIFN-*γ in vitro* treatment as previously described [18]. MDMs from the patient displayed a normal capacity to phagocytose* M. tuberculosis* in comparison to healthy controls (Figure 2(a)), which was not significantly increased by rhIFN-*γ*. On the other hand, MDMs from the patient failed to control the proliferation of* M. tuberculosis* in comparison with the control's MDMs, a functional defect which was improved by rhIFN-*γ* (Figures 3(a) and 3(b)).

## 4. Discussion

Host immune response against* M. tuberculosis* is mediated by IL-12/IFN-*γ* axis. In the process of control of the infection by mycobacteria, IL-12, IL-6, and TNF-*α* seem to have a primordial function. These cytokines act in synergy with IFN-*γ*, stimulating the production of oxidative burst, thus mediating the tuberculostatic function of macrophages, and also stimulating the migration of immune cells to the infection site, contributing to granuloma formation, which controls the disease progression. IFN-*γ* is the main cytokine involved in the immune response against mycobacteria, and its major function is the activation of macrophages, allowing them to exert their microbicidal role functions [20]. Effective TB control requires a deeper understanding of the impact of the immune response underlying the relative success of drug-resistant strains; therefore, to better understand whether impaired macrophage's function contributes to the insufficient mycobacterium control in MDR-TB patients, we investigated the differentiation and function of MDMs from patients with MDR-TB and* in vitro* function of IFN-*γ* in MDMs culture have been characterized. We found that MDMs from these patients have reduced cytokine as well as oxidative burst production and impaired expression of IFN-*γ*R1 compared to the MDMs from normal controls, while rhIFN-*γ* was able to significantly improve all observed defects. More importantly, rhIFN-*γ* also improved the defective ability of patient macrophages to control the growth of* M. tuberculosis*.

The defective production of cytokines such as IL-12, IL-6, and TNF-*α* by patient's macrophages suggests that in MDR-TB not only IL-12/IFN-*γ* axis is abnormal but also anomalies in the initiation of inflammatory responses are in accordance with previous results in human and mice [21, 22]. Macrophages from our patients with susceptibility to mycobacterial diseases showed normal ability to phagocytose* M. tuberculosis *which may be due to normal TLR expression on patient's MDMs, however failing to control the intracellular growth of* M. tuberculosis* in comparison to those from healthy subjects. It is in accordance with Carranza and collaborators who previously showed that macrophages from TB patients were less capable of controlling* M. tuberculosis* growth [23]. The mechanism of impaired growth control of* M. tuberculosis* by our patients MDMS still needs further molecular and genetic characterization for searching genetic defects in these patients in which the MDR-TB will not be only cause of poor responsiveness to TB treatment because our cohort is comprised of consanguineous families and presenting susceptibility to only mycobacteria. The idea that life-threatening infectious diseases occurring in otherwise healthy individual, during the course of primary infection, may result from single-gene inborn errors of immunity that is gaining ground [24–26]. One of the most thoroughly investigated pediatric syndromes is Mendelian susceptibility to mycobacterial disease (MSMD) (OMIM 209950), a rare disorder (affecting about 1 in 100 000 individuals), predisposing individuals to severe clinical disease upon infection with weakly virulent mycobacteria, including Bacille Calmette-Guérin (BCG). These patients are frequently susceptible to* Salmonella* and* M. tuberculosis*. However, the genetic etiologies of many patients with MSMD remain to be identified [27, 28]. Moreover, based on our data, the possible mechanisms may be defective cytokines and oxidative burst production as well as reduced surface expression molecules which may be attributed to macrophages dysfunction in our patients.

The optimal* M. tuberculosis* growth control by macrophages requires cytokine activation.* In vitro* models of macrophage activation for the killing of* M. tuberculosis* are rather artificial, and therefore the exact conditions for optimal activation remain unknown [29]. The mechanism by which IFN-*γ* improves the functional defects of phagocytes occurs at both the level of a progenitor cell and mature cells [30]. This cytokine enhances the oxidative burst response, but, on the other hand, IFN-*γ* has been shown to contradictorily improve neutrophil microbicidal killing through mechanisms other than enhanced oxidative activity [31]. IFN-*γ* is an important cytokine which plays multifarious roles in different parts of the immune system. It is involved in the process of generating, sustaining, and regulating the cells of the innate and adaptive arms of the immune system. Macrophages activated by IFN-*γ* increased pinocytosis, receptor-mediated phagocytosis, and microbial killing ability against mycobacteria. As we studied a small cohort, the continuation of this research line is required to study increased number of patients with MDR-TB in order to study comprehensively MDM mechanisms of controlling* M. tuberculosis* growth. In addition, large randomized controlled trials have been already performed showing that adjuvant therapy using IFN-*γ* might be beneficial to TB patients [19].

Taking together our data suggesting that the treatment of patients with poor responsiveness to TB therapy may be needed to include IFN-*γ* as adjuvant therapy after full characterization of immunopathologic mechanisms in these and in other more multidrug-resistant TB patients.

## Figures and Tables

**Table 1:** Gender, age, and consanguinity of MDR-TB patients.

Pt	Gender	Diagnosis (years)	MDR-TB	Consanguinity affected	Family member
P1	Male	20	+	+	+
P2	Male	30	+	+	+
P3	Male	36	+	+	−
P4	Male	40	+	+	−
P5	Male	19	+	+	−
P6	Female	18	+	+	+
P7	Male	23	+	+	+
P8	Female	34	+	+	−
P9	Male	27	+	+	−
P10	Female	43	+	+	+

Symbols: +, present; −, absent.

## Abbreviations

MDMs: monocyte-derived macrophages,
rhIFN-*γ*: recombinant human interferon-gamma,
TLR: toll-like receptor,
TB: Tuberculosis.
